# Avelumab plus axitinib for translocation renal cell carcinoma: A case series and literature review

**DOI:** 10.1002/iju5.12685

**Published:** 2023-12-27

**Authors:** Kenta Takahashi, Renpei Kato, Daiki Ikarashi, Tomohiko Matsuura, Shigekatsu Maekawa, Mitsugu Kanehira, Ryo Takata, Jun Sugimura, Takaya Abe, Wataru Obara

**Affiliations:** ^1^ Department of Urology Iwate Medical University Shiwa‐gun Iwate Japan

**Keywords:** avelumab, axitinib, immune checkpoint inhibitors, renal cell carcinoma, translocation renal cell carcinoma

## Abstract

**Introduction:**

Patients with translocation renal cell carcinoma (tRCC) have a poor prognosis without standardized treatment.

**Case presentation:**

The first case was of a 72‐year‐old woman who underwent robot‐assisted partial nephrectomy for a left renal tumor and was pathologically diagnosed with tRCC. Recurrence was observed in the left retroperitoneal soft tissue. After treatment with avelumab–axitinib, continued progression‐free survival was confirmed at the 90‐week follow‐up. The second case was of a 41‐year‐old woman referred to our hospital and presented with translocation renal cell carcinoma metastasis to a para‐aortic lymph node. After treatment with avelumab–axitinib, continued progression‐free survival was confirmed at the 43‐week follow‐up.

**Conclusion:**

The outcomes of these cases indicate that avelumab–axitinib therapy has a long‐term antitumor effect in some patients with tRCC.

**Abbreviations & Acronyms**
CT: computed tomography
IHC: immunohistochemical
IMDC: International Metastatic RCC Database Consortium
IO: immune‐oncology
irAE: immune‐related adverse event
non‐ccRCC: non‐clear‐cell RCC
NRF2: nuclear factor‐erythroid 2‐related factor 2
PD1/L1: programed cell death protein 1/L1
PFS: progression‐free survival
PR: partial response
RCC: renal cell carcinoma
TKI: tyrosine kinase inhibitor
tRCC: translocation renal cell carcinoma

**Keynote message**

The treatment of tRCC with avelumab–axitinib achieved good tumor reduction and can be considered a long‐term therapy because adverse events can be controlled easily.

## Introduction

The incidence rate of translocation tRCC in adults is 1%–5% of all RCC cases. Translocation RCC has a very poor prognosis.^1^ For advanced non‐ccRCC, including tRCC, no standardized treatment exists, although some reports have shown the efficacy of IO combination therapy.

This case report presents the cases of two women with tRCC treated with avelumab–axitinib who showed relatively good tumor reduction. These results indicate that avelumab–axitinib treatment can be a long‐term therapy for tRCC.

## Case presentation

**Fig. 1:**
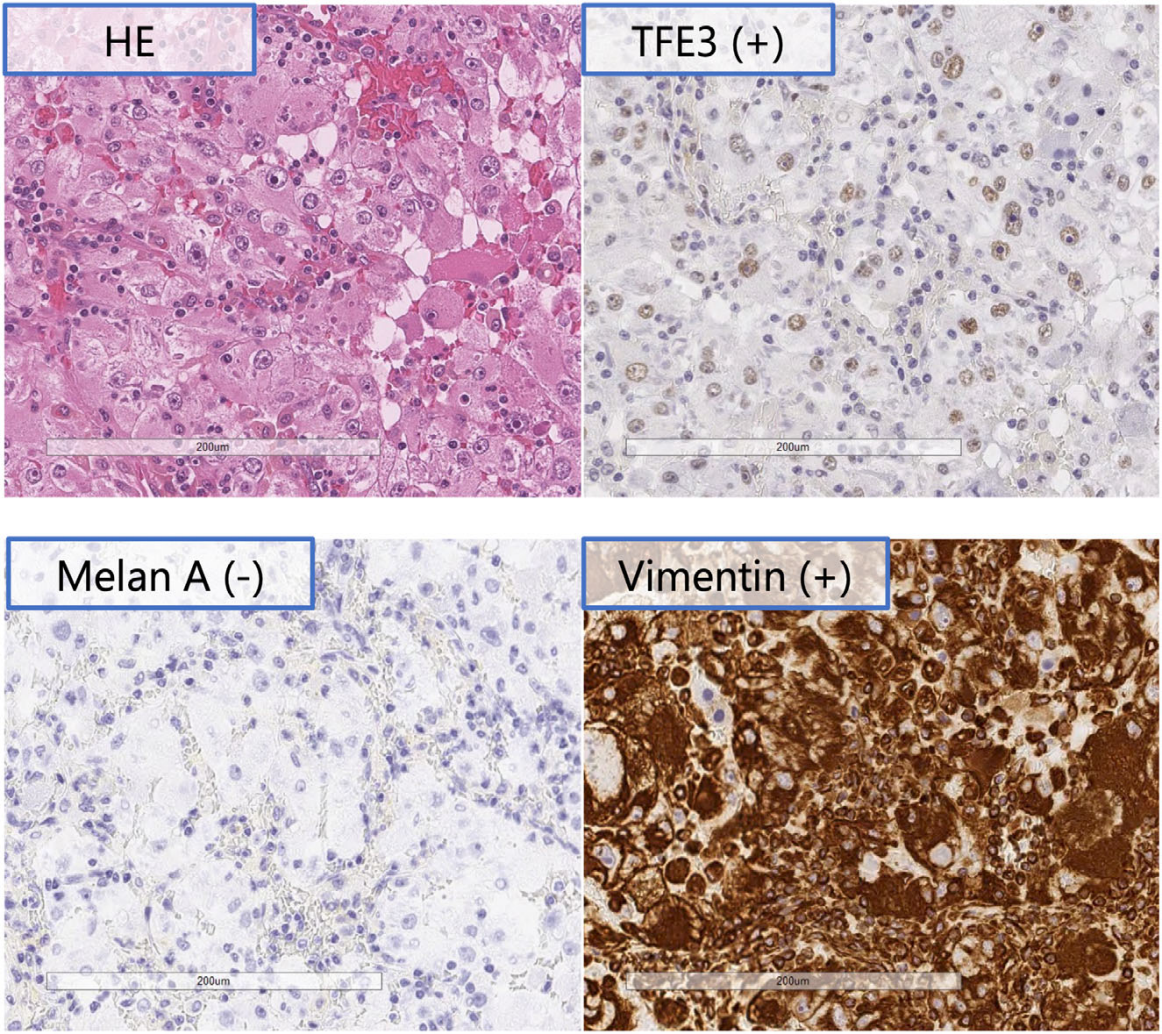
IHC analysis in Case 1. Hematoxylin and eosin staining shows foci of proliferating tumor cells; TFE3 positivity suggested tRCC, MelanA negativity ruled out TEFB‐positive tRCC, and Vimentin negativity ruled out anaplastic renal carcinoma.

The first case was of a 72‐year‐old woman. CT revealed a 26 × 26‐mm^2^ enhancing mass with a contrast effect on the left inferior pole of the kidney. A left renal tumor was found, and a robotic‐assisted partial left nephrectomy was performed using a retroperitoneal approach. IHC evaluation revealed that the tumor was a‐methyl acyl coenzyme A racemase+, vimentin+, transcription factor E3+, melan‐A+, and cytokeratin 7− (Fig. 1). Based on these findings, tRCC was the final diagnosis, a TFE3‐related RCC, at the pathological stage T1aN0M0.

**Fig. 2:**
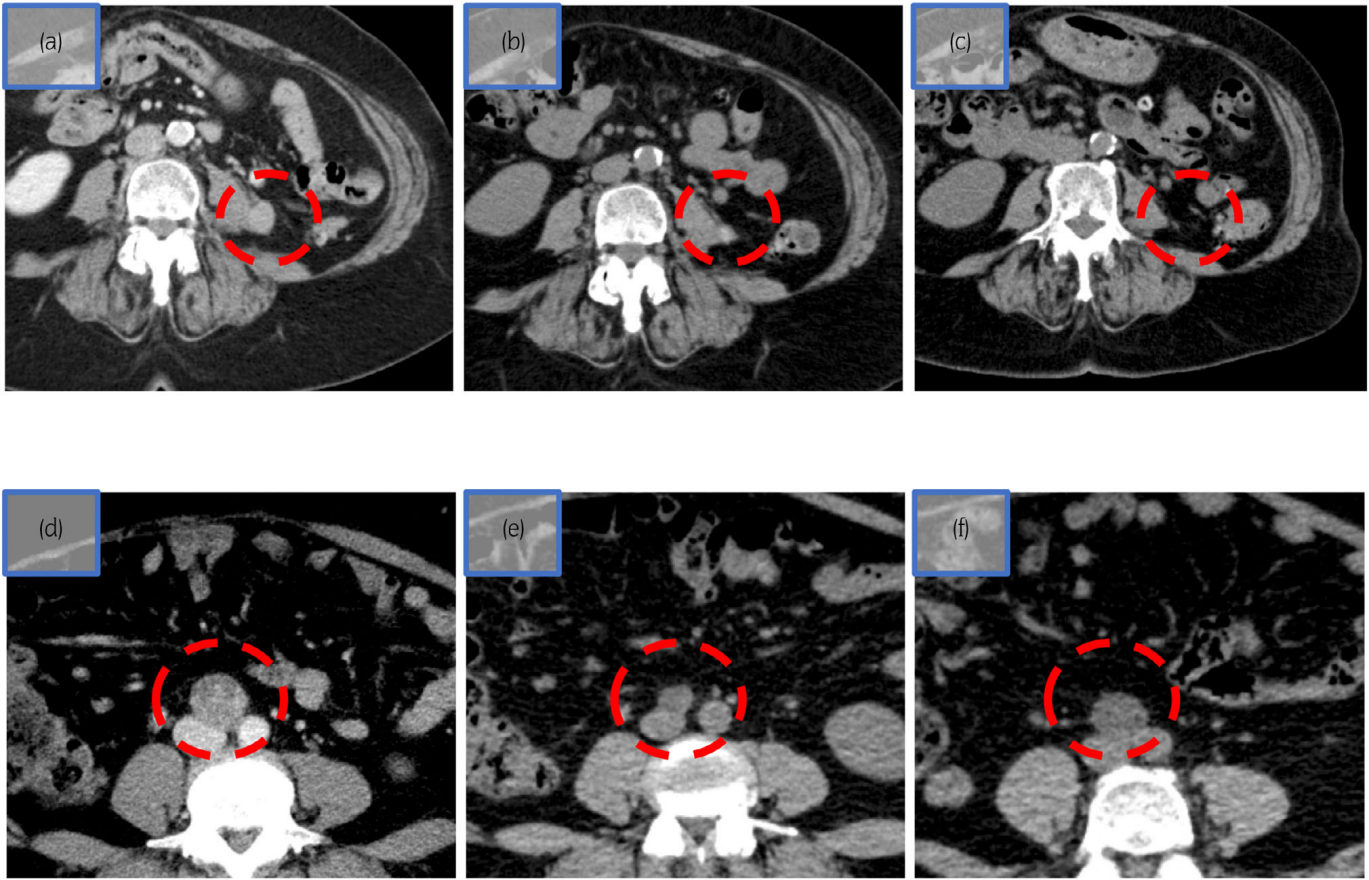
CT. (a–c) is first case and (d–e) is second case. (a) Twenty‐four months after surgery, retroperitoneal soft tissue tumor had a maximum diameter of 15 mm. (b) Thirty weeks after starting avelumab and axitinib, tumor size decreased to a maximum diameter of 10 mm, at 33% reduction rate. (c) Ninety weeks after starting avelumab and axitinib, tumor had almost disappeared (2 mm maximum diameter), at 86% reduction rate. (d) Para‐aortic lymph node with a maximum diameter of 32 mm. (e) Twenty‐five weeks after starting avelumab and axitinib, tumor size decreased to a maximum diameter of 13 mm, at 59%reduction rate. (f) Forty‐two weeks after starting avelumab and axitinib, tumor increased to a maximum diameter of 18 mm.

**Fig. 3:**
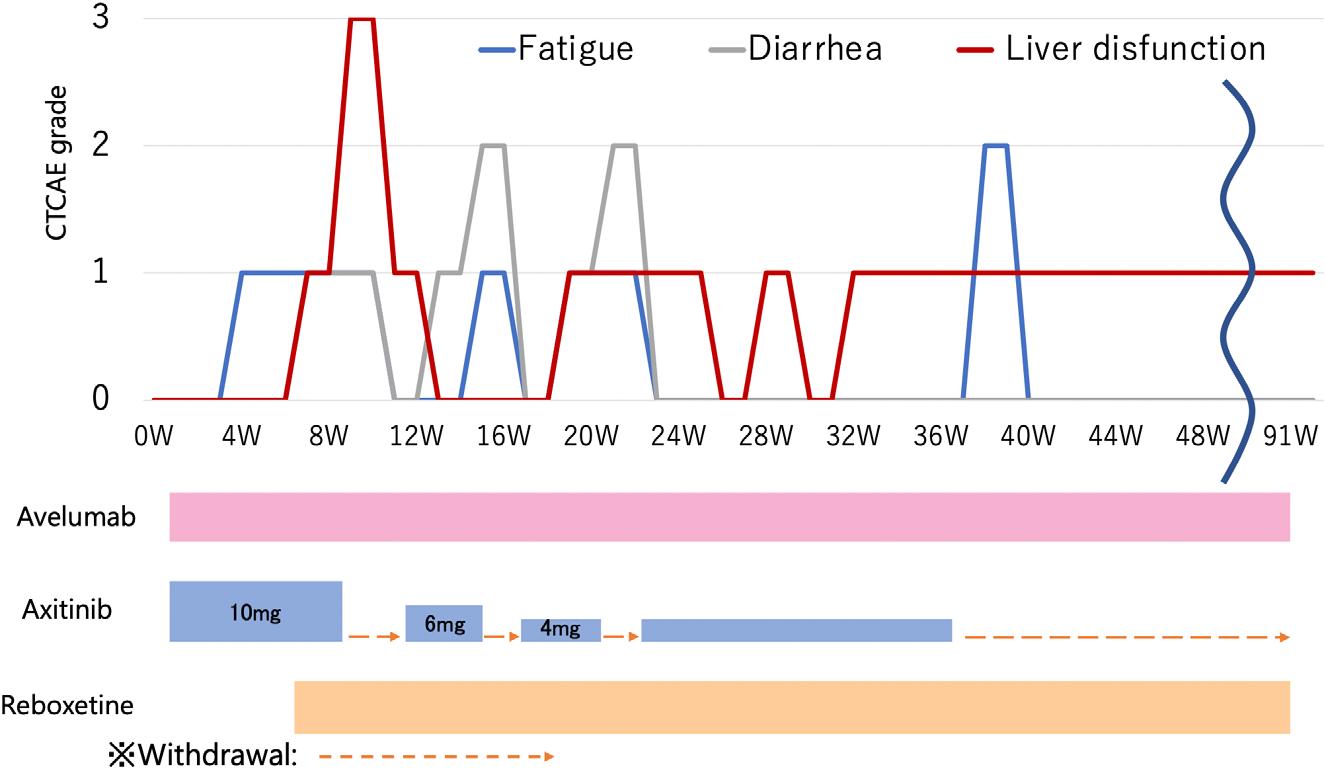
Treatment course in Case 1. Vertical axis: Common Terminology Criteria for Adverse Events grade of adverse effects; horizontal axis: progression is represented by blue line for fatigue, gray line for diarrhea, and red line for liver dysfunction; Axitinib was withdrawn due to liver dysfunction, avelumab was continued, and the metastases remained reduced.

A retroperitoneal soft tissue tumor appeared 26 months after surgery (Fig. 2a). The IMDC classification at the time of postoperative recurrence was “favorable.” Treatment with avelumab (10 mg/kg) and axitinib (10 mg/day) combination was initiated. At the 4‐week follow‐up, liver dysfunction (Common Terminology Criteria for Adverse Events [CTCAE ver.5.0] grade 3) occurred; thus, axitinib was discontinued (Fig. 3). At the 11‐week follow‐up, the patient's liver dysfunction improved, and consequently, axitinib was resumed at 6 mg/day dosage. Hypothyroidism (CTCAE grade 2) occurred as an irAE but was controlled with reboxetine. At the 30‐week follow‐up, the tumor was judged as a PR (the New Response Evaluation Criteria in Solid Tumors [RECIST] v1.1) (Fig. 2b). Because of the subsequent onset of fatigue (CTCAE grade 2) and liver dysfunction (CTCAE grade 1), axitinib was discontinued, and only avelumab was continued. At the 90‐week follow‐up, the tumor size continued to decrease (Fig. 2c).

The second case was of a 41‐year‐old woman referred to our hospital with tRCC metastasis diagnosis, which was TFE3‐related RCC, to the para‐aortic lymph node. The patient was diagnosed with left kidney cancer without metastasis in her previous hospital, which performed a laparoscopic nephrectomy. IHC evaluation revealed that the tumor was AMACR+, TFE3+, CA19‐9+, PAX3+, CK7−, and cathepsin K−, and the pathological diagnosis was tRCC at the pathological stage T3aN0M0. Para‐aortic lymph node enlargement (32 × 20 mm^2^) appeared 26 months after surgery (Fig. 2d). Since imaging studies did not reveal an obvious primary tumor, the diagnosis was lymph node metastasis of tRCC. The IMDC classification during postoperative recurrence was “favorable risk” (not applicable). At this point, the patient was referred to our hospital due to relocation.

**Fig. 4:**
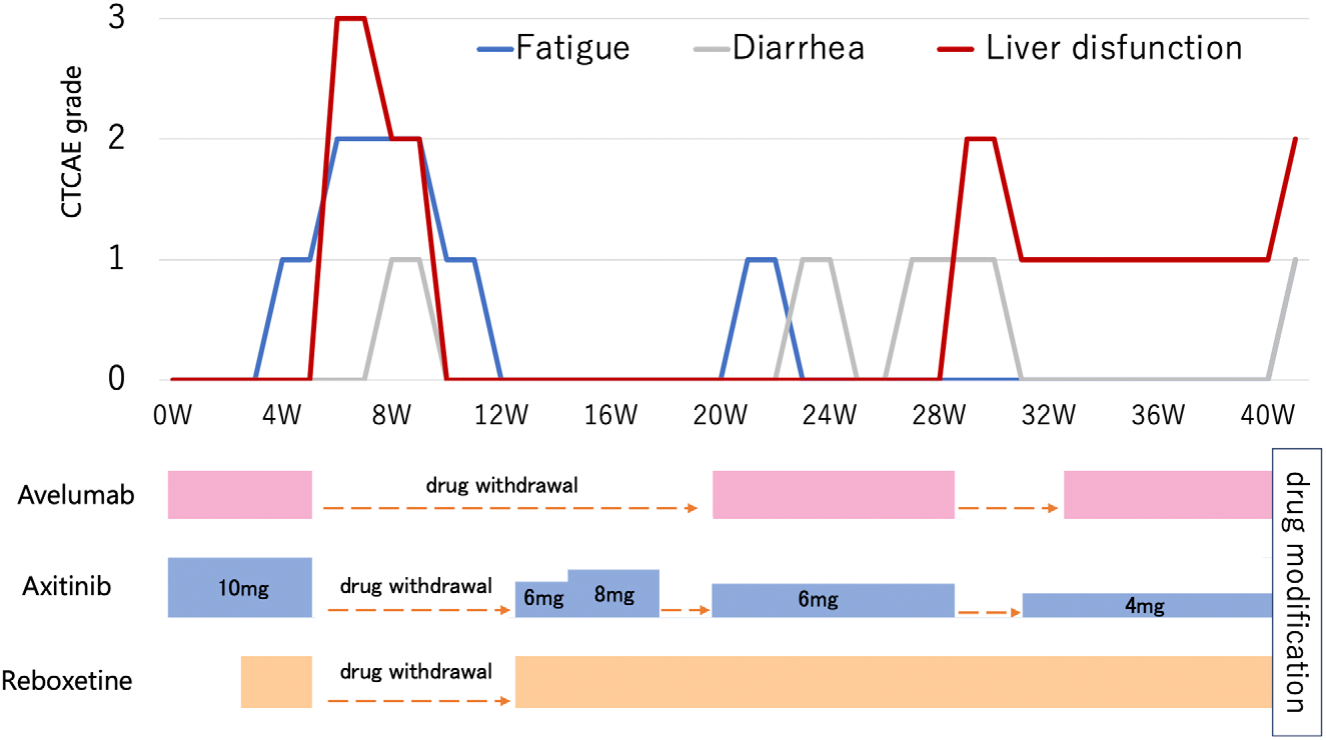
Treatment course in Case 2. Vertical axis: Common Terminology Criteria for Adverse Events grade of adverse effects; horizontal axis: progression is represented by blue line for fatigue, gray line for diarrhea, and red line for liver dysfunction; 43 weeks after starting avelumab and axitinib, treatment was modified due to hepatic dysfunction and increased.

One month after the metastasis onset, avelumab (10 mg/kg) and axitinib (10 mg/day) combination therapy was initiated. At the 6‐week follow‐up, therapy was discontinued because of destructive thyroiditis (CTCAE grade 2) and liver dysfunction (CTCAE grade 3) (Fig. 4). The patient's liver dysfunction improved with drug withdrawal, and hypothyroidism after destructive thyroiditis improved after treatment with reboxetine. Treatment with avelumab (10 mg/kg) and axitinib (6 mg/day) combination was resumed. At the 25‐week follow‐up, the maximum diameter of the para‐aortic lymph node was reduced and judged as PR using RECIST (Fig. 2e). At the 40‐week follow‐up, CT revealed disease progression according to RECIST (Fig. 2f). At present, cabozantinib is initiated as a second‐line therapy.

## Discussion

The standard first‐line treatment recommended for advanced RCC is IO combination therapy.^2^, ^3^, ^4^, ^5^, ^6^, ^7^ The efficacy of IO or tyrosine kinase inhibitor (TKI) monotherapy for tRCC has been indicated.^8^, ^9^, ^10^, ^11^ PFS for treatment with TKI monotherapy is reportedly 3–7 months, and PFS for treatment with IO monotherapy is reportedly 2.5 months. To date, only a few reports have shown the efficacy of IO combination therapy for advanced tRCC. A phase II study of nivolumab and cabozantinib for non‐ccRCC revealed efficacy.^12^ However, the study only assessed two cases of tRCC, and the usefulness of IO combination therapy in treating tRCC was not established.

Recently, the benefits of IO combination therapy for tRCC were explored through the detailed evaluation of genetics and the tumor immune environment.^1^ In this study, a detailed genetic analysis of tRCC was performed, which revealed the fusion partners of TFE3, TFEB. In our cases, tRCC was diagnosed by IHC, and no genetic analysis was performed. The abovementioned study also analyzed the efficacy of systemic therapy for tRCC. The expression of NRF2 was elevated in tRCC compared with other histological types. NRF2 is a transcription factor related to oxidative stress, and its expression has been associated with TKI resistance.^13^ The tumor immune environment revealed that the density of CD8+ PD1/L1+ T cells in the tumor, possibly involved in sensitivity to PD1/L1 inhibitors, was high. This was because the tumor neoantigen possibly recruits T cells in tRCCs derived from the fusion junction.^1^ They further compared the efficacy of atezolizumab–bevacizumab with sunitinib using cases of tRCC from IMmotion 151 trial, revealing that atezolizumab–bevacizumab significantly extended PFS. Given this evidence, the efficacy of IO combination therapy in treating tRCC is suggested.

Treating tRCC with avelumab–axitinib can be long term because AEs are easily controlled. The rate of severe adverse effects in phase III trials was higher for IO–TKI therapy^3^, ^4^, ^5^, ^6^ than IO–IO.^7^ This is probably due to most AEs of IO–TKI therapy being similar to TKI profiles. Regarding the control of AEs for TKIs, the half‐life of axitinib is the shortest among other TKIs; this indicates that the control of AEs is easier with the use of axitinib through drug withdrawal and dose reduction.^14^ Furthermore, the rate of steroid use for irAEs caused by avelumab and axitinib is the lowest among other IO combination therapies. Furthermore, concerning PD‐1 or PD‐L2, the rate of irAEs caused by avelumab is possibly low because ligands remain in place.

## Conclusion

This case series described two patients with tRCC who were treated with avelumab–axitinib and experienced good tumor reduction. Furthermore, treatment with avelumab–axitinib can be a long‐term therapy for tRCC with few or easily controlled AEs.

## Author contributions

Kenta Takahashi: Conceptualization; writing – original draft. Renpei Kato: Conceptualization; writing – review and editing. Daiki Ikarashi: Data curation. Tomohiko Matsuura: Data curation. Shigekatsu Maekawa: Supervision. Mitsugu Kanehira: Supervision. Ryo Takata: Supervision. Jun Sugimura: Supervision. Takaya Abe: Supervision. Wataru Obara: Supervision; writing – review and editing.

## Conflict of interest

The authors declare no conflicts of interest.

## Approval of the research protocol by the Institutional Reviewer Board

This study was approved by the Ethics Committee of Iwate Medical University (MH2021‐0679).

## Informed consent

Informed consent was obtained from the patients for the publication of this case report and the accompanying images.

## Registry and the Registration No. of the study/trial

Not applicable.
